# An intensive multimodal group programme for patients with psychotic disorders at risk of rehospitalization: a controlled intervention study

**DOI:** 10.1186/s12888-019-2229-x

**Published:** 2019-08-05

**Authors:** Mark H. de Jong, André I. Wierdsma, Arthur R. Van Gool, Cornelis L. Mulder

**Affiliations:** 1 Yulius Mental Health, PO Box 1001, 3300 BA Dordrecht, The Netherlands; 2 Epidemiological and Social Psychiatric Research Institute (ESPRi), Department of Psychiatry, Erasmus University Medical Centre, Postbus 2040, 3000 CA Rotterdam, The Netherlands

**Keywords:** Community mental healthcare, Severe mental illness, Group therapy, Psychotic disorder, Psychiatric admission, Mental healthcare costs

## Abstract

**Background:**

On the basis of earlier experiences in Germany and England, we developed an intensive multimodal group programme (FACT Plus) for psychotic-spectrum patients. By combining it with regular Flexible Assertive Community Treatment (FACT) (care as usual), we intended to reduce psychiatric rehospitalizations and mental healthcare costs.

**Methods:**

We included adult patients (>18 years) with a psychotic spectrum disorder who had had at least one psychiatric admission in the 2 years before inclusion. FACT Plus was delivered weekly for 9 months. The intervention group was recruited in northern Rotterdam (the Netherlands), and the control group was recruited in southern Rotterdam. The primary outcome measure was length of stay (LOS) and the secondary outcome measures were mental healthcare costs and compulsory admissions.

**Results:**

We included 52 patients in the intervention group and 61 patients in the control group. During the 12-month observation period, the mean LOS per patient was 15.2 (intervention group) and 34.6 (control group). This represents a difference of 19.4 days (56.1%). This result was statistically significant (B = −.859, SE = .497, *p* = .042) in a regression model correcting for baseline differences between the groups. Mean total mental healthcare costs per patient were €21,098 in the intervention group) versus €25,054 in the control group, a difference of about €4000 per patient (16%). In addition, there were zero compulsory admissions in the intervention group and nine in the control group.

**Conclusions:**

After the addition of FACT Plus to regular FACT, psychiatric LOS was substantially lower in the intervention group than in the control group. This result was accompanied by a limited reduction in mental healthcare costs.

## Background

The success of community-based treatment as a means of preventing the admission of patients with severe mental illness [1, 2] remains disappointing. The shortcomings of this approach are demonstrated by the number of patients with a history of frequent voluntary or involuntary psychiatric hospitalization [3–5]. Although assertive community treatment (ACT) [6] has been found to have some positive effect for patients with severe mental illness with regard to hospitalization, social outcome, and retention in care, these effects are slight and the quality of evidence is moderate at best [7].

Early this century, a variant of ACT called Flexible Assertive Community Treatment (FACT) was developed in the Netherlands. While FACT provides routine community mental health services when possible, it offers intensive ACT when necessary [8, 9]. But whatever the exact treatment programme, a group of patients with severe mental illness remains, many of whom are frequently admitted to psychiatric hospital [3, 5]. To reduce readmissions in this subgroup of severe mental illness patients, we therefore sought an intervention that could be implemented in addition to FACT.

In Germany, positive results were shown by an intervention known as the Munich Psychosis Information Project [10], which used an intensive multimodal group programme. Its interventions included psycho-education focussing on the importance of antipsychotic drug adherence and family treatment. Rehospitalizations during 12-month follow-up were approximately 50% lower in the intervention group than in the control group. Completer analysis showed this result to be significant, whereas the result of intention-to-treat-analysis did not reach significance. This important difference between completer analysis and intention-to-treat-analysis may be due to biases, e.g. selective drop out, so this result should be interpreted with caution. A post hoc study with a seven-year follow-up found that, overall, hospitalization rates, adherence, and course of illness were still better in the intervention group than in the control group [11].

This German programme was also adopted and implemented in England as the Maintaining Adherence Programme, where, in a pre-post design, it significantly reduced in-patient bed days by 42% in the intention-to-treat analysis and by 51% reduction in the completer analysis [12]. The same study also showed a significant cost reduction and very high patient and staff satisfaction.

However, neither of these two studies provided definitive answers.

## Methods

### Aim

Unaware of any other group-format interventions associated with reduced admissions in patients with severe mental illness at risk of rehospitalization, we aimed to replicate the German and English findings and decided to develop a Dutch adaptation of these programmes, which we implemented in Dutch FACT teams. We hypothesized that combining this intensive multimodal group programme with regular FACT in a group of patients at risk of rehospitalization would reduce voluntary and involuntary hospitalizations, and also overall mental healthcare costs, more than FACT as usual.

### Intervention

Our intensive multimodal group programme, which we called FACT Plus, added several interventions and activities to FACT. It consisted of weekly 90-min group sessions for as much as 9 months, plus two-weekly follow-up sessions for the next 3 months. The programme involved psychoeducation, recovery planning, shared decision making, composing a crisis plan, drug-therapy adherence monitoring, resocialization activities, family interventions, and sport and lifestyle interventions. To monitor the status and to promote the progress of the programme, we used a digital support platform that sent automatic text messages to remind participants of the sessions and also supported the process for the participants, their family members, and the professionals.

**Fig. 1:**
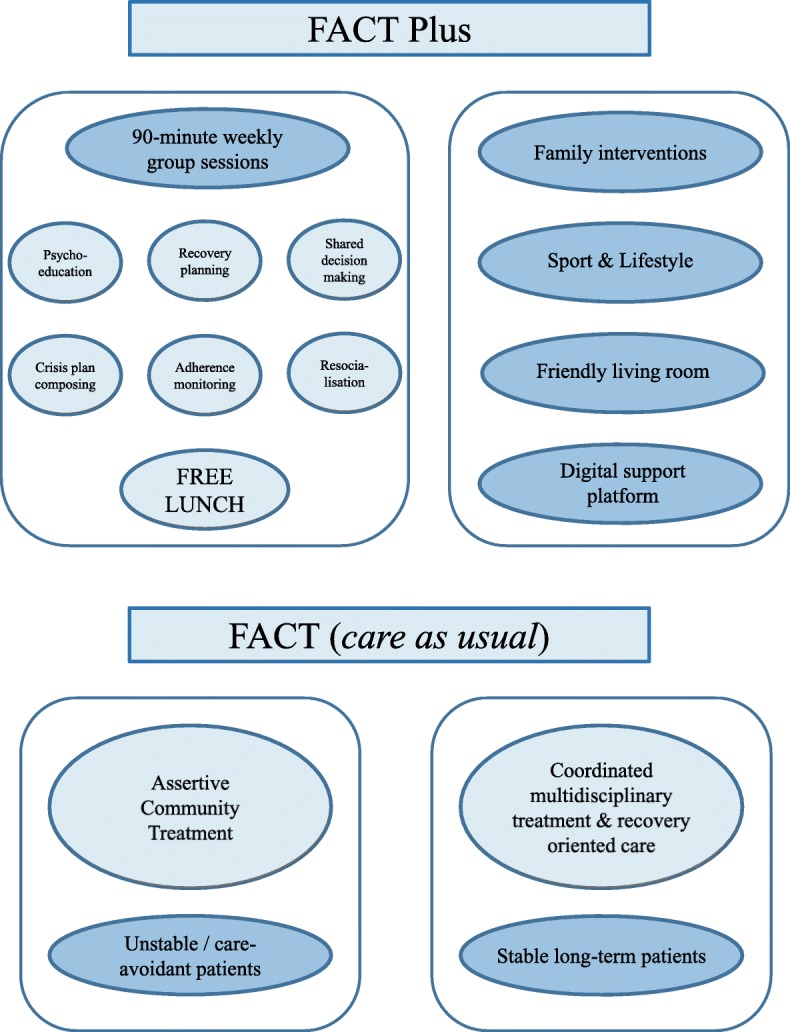
Core points of FACT Plus and FACT

FACT Plus was carried out in a friendly living-room like atmosphere that was designed to denote accessibility and hospitality. Importantly, a free lunch was offered after every morning session and before every afternoon session. Participants and professionals did the shopping for lunch together, and also prepared the food. In this way, lunch was itself an intervention, as it was intended to support resocialisation and to educate the participants about healthy food and cost-conscious shopping. Along with the programme, regular FACT care as usual was continued (see also Fig. 1).

### Care as usual

Care as usual consisted of FACT teams providing assertive community treatment for unstable and care-avoiding patients, and coordinated, multidisciplinary treatment and recovery-oriented care for stable long-term patients [8, 9]. Per team, the mean number of patients is 200, all of whom are treated for severe mental illness, mainly schizophrenia and other psychotic disorders. To promote comparable quality of care in all FACT teams, teams are regularly audited on the basis of well-described standards and criteria.

### Design

This pilot study had a quasi-experimental controlled study design. Participants received the FACT Plus programme in combination with FACT. The control patients received regular FACT only (i.e. care as usual). On the basis of the intention-to-treat principle, all patients who entered the study were included in the outcome analyses, including those who dropped out of the intervention early. According to this design, we conducted a prospective intervention study with an intervention group and a control group. The observation period for both groups was 12 months. While the observation period for the participants started at inclusion, a fixed observation period was chosen for the controls after all eligible patients had been identified (1 April 2016–31 March 2017).

### Outcome measures

The primary outcome measure was psychiatric hospitalization, which was expressed as the length of stay (LOS) in days. Secondary outcome measures were the number of hospitalizations, the mental healthcare costs, and the number of compulsory admissions. Mental healthcare costs consisted of actual costs for outpatient care, including the expended staff time for the group sessions in the intervention group, inpatient care, and additional costs for the programme, such as lunches and materials in the intervention group. Basic demographic, clinical and outcome data was collected for both groups.

### Setting

Participants and controls were recruited from the FACT teams at the Parnassia Psychiatric Institute, a mental health organization in the Netherlands’ greater Rotterdam area. FACT Plus was delivered to patients living in northern Rotterdam, whereas patients in the control group were selected in southern Rotterdam. While the organization, procedures, staff, and patients of the FACT teams in both parts of the city were very comparable, they were clearly separated geographically by the river Maas. Professionals involved in the intervention programme did not work in the control-group teams and vice versa.

One psychiatric hospital on either side of the river Maas was available for all patients included in the study. While patients in the intervention group were admitted to the hospital in northern Rotterdam, some patients in the control group were allocated to the northern hospital and some to the southern hospital. The two hospitals used identical admission and discharge criteria, which were documented in a service-delivery agreement covering all psychiatric hospitals within the region. The annual mean length of stay in the research period was comparable between the two hospitals.

### Participants

Adult patients (> 18 years) in the regular treatment of a FACT team were eligible for the study if they had been admitted to a psychiatric hospital at least once in the 2 years before inclusion. This admission criterion was applied in order to select patients at the highest risk of rehospitalization and those who accounted for the highest mental healthcare costs. We included only patients with a diagnosis, according to the DSM-IV, [13] in the psychotic spectrum (schizophrenia, schizoaffective disorder, psychotic disorder not otherwise specified (NOS), and delusional disorder). Patients with severe learning disabilities, severe conduct disorders, and severe language barriers were excluded. We included 52 patients in the intervention group and 61 patients in the control group.

Table 1 summarizes the baseline characteristics of the two groups. While, overall, participants and controls had identical characteristics, more participants in the intervention group had schizophrenia (71.2% versus 50.8%, respectively**)**, whereas more participants in the control group had psychotic disorder NOS (34.4% versus 17.3%). And while more patients in the intervention group used depot medication, a slightly higher number of patients in the control group used no antipsychotic medication.

**Table 1:** Demographic and clinical characteristics of participants and controls

Variable	Participants N (%)	Controls N (%)
N	52	61
Age (mean, yrs)	37.5	42.7
Male gender	33 (63.5)	40 (65.6)
Classification (DSM-IV)		
Schizophrenia	37 (71.2)	31 (50.8)
Schizoaffective disorder	6 (11.5)	8 (13.1)
Psychotic disorder NOS	9 (17.3)	21 (34.4)
Delusional disorder	0 (0.0)	1 (1.6)
Antipsychotics		
No	1 (1.9)	8 (13.1)
Oral	30 (57.7)	34 (55.7)
Clozapine	7 (13.5)	11 (18.0)
Depot	21 (40.4)	19 (31.1)
Dx of addiction (except nicotine)	30 (57.7)	30 (49.2)

### Ethical issues and data processing

Our study was approved by the Medical Ethics Committee at Leiden University Medical Centre. All participants in the intervention group signed the informed consent form prior to inclusion. However, as the control patients were not subjected to any assessment or study intervention, the medical ethics committee waived their obligation of informed consent. All patient-related data concerning participants and control group were also carefully anonymized, each patient’s data coded with a research number. The hospitalization data and mental healthcare costs of each group were collected from the electronic patient registration system of the Parnassia Psychiatric Institute. Information on whether participants or controls had been hospitalized in other mental health inpatient care facilities was gathered by the first author (MJ), who scrutinized all patient files.

### Statistical analysis

Descriptive statistics were used to explore the demographic and clinical differences between the intervention and control groups. In view of the hypothesis that the intervention would lead to reduced LOS, statistical tests were one-sided, with the statistical significance level set at 5%. Due to the small sample size, correction for multiple testing was waived. To evaluate the treatment effect, generalized linear models were constructed in which we corrected for possible baseline differences between the groups. To investigate the difference in LOS, we used the negative binomial distribution with loglink, which concerns count data with a high number of zeroes. The gamma distribution was then applied to evaluate differences in right-skewed, positive and continuous data on mental healthcare costs. All statistical analyses were conducted using SPSS software (IBM SPSS version 24.0).

## Results

### Primary outcome

During the 12-month observation period in the intervention group, the mean LOS was 15.2 days per patient versus 34.6 days in the control group – a difference of 19.4 days (56.1%) in LOS between the two groups. After correction for potential differences between the groups in terms of age, gender, diagnosis, antipsychotics and drug abuse, the final model showed a statistically significant difference in LOS (B = -.859, SE = .497, *p* = .042). Table 2 summarizes the outcomes and sensitivity analyses.

**Table 2:** Hospitalization days and total mental healthcare costs (summary of outcomes and sensitivity analyses)

Outcome	Intervention group	Control group	Group parameter
Hospitalization days (Mean and SD)	15.2 (32.2)	34.6 (76.4)	
- Adjusted (estimated mean and SE)^a^	14.7 (5.4)	34.6 (11.7)	-.859 (*p* = .042)
- Excluding baseline hospitalized patients	14.7 (5.4)	25.5 (8.5)	-.554 (*p* = .13)
- Excluding outlier	13.6 (4.8)	25.1 (8.7)	-.614 (*p* = .10)
Total mental healthcare costs € (Mean and SD)	21,098 (18,659)	25,054 (39,196)	
- Adjusted (estimated mean and SE)^a^	21,501 (3262)	26,152 (3820)	−.196 (*p* = .172)
- Excluding baseline hospitalized patients	22,895 (3317)	18,070 (2675)	.237 (*p* = .125)
- Excluding outlier	20,249 (2890)	20,778 (2989)	−.026 (*p* = .448)

^a^Model includes casemix factors (medication formulation, psychosis, drug abuse, gender and age grand mean centered); Wald-test, one-sided *P*-values

### Secondary outcomes

The number of hospitalizations did not differ between the two groups (39 in the intervention group versus 38 in the control group). During 12-month follow-up, the mean total mental healthcare costs per patient were €21,098 in the intervention group (including the costs of FACT Plus) versus €25,054 in the control group, a difference of about €4000 per patient (16%). In a regression analysis this difference in costs was not statistically significant (B = -.196, SE = .207, *p* = .172). In the model we used identical corrections for potential differences between the intervention and control groups, as we did for the primary outcome. The additional costs for the FACT Plus program for lunches and materials added €135 on average to the total per patient, which is a very small proportion of the mean total costs of €21,098 per patient in the intervention group.

During follow-up, there were no compulsory admissions in the intervention group versus nine in the control group. These numbers were too small for further statistical analysis.

### Sensitivity analyses

Despite fulfilling all the inclusion criteria, five patients in the control group turned out on the first day of the chosen observation to have been hospitalized. During the follow-up period, another patient in the control group was hospitalized for more than 300 days (outlier). After exclusion of the patients who were in hospital at the start of the observation period, sensitivity analyses regarding the number of hospitalization days showed that the difference in LOS between the two groups was somewhat smaller (B = −.554, SE = .492, *p* = .13). Otherwise, when we excluded the outlier, again the difference in LOS (B = −.614, SE = .489, *p* = .10) changed only slightly.

With regard to costs, the exclusion of the patients hospitalized at baseline, the model showed a difference in costs in favour of the control group (B = .237, SE = .206, *p* = .125). Otherwise, when we excluded the outlier, there was a small difference in costs in favour of the intervention group (B = -.026, SE = .197, *p* = .448).

## Discussion

### Main results

Our results showed that mean length of stay (LOS) 56.1% lower for patients who had participated in the FACT Plus intensive multimodal group programme than it was for patients in the control group. After the 12-month observation period, we observed a limited saving of about €4000 per patient with regard to the total mental healthcare costs in the intervention group compared to the control group. The outcomes may be the product of various factors within an assertive and integrated approach, especially a welcoming environment, psychoeducation, the promotion of adherence, the involvement of families, and the strong fellow-feeling between the patients themselves.

As the number of admissions did not differ significantly between the two groups, we might state that while similar numbers of patients from the two groups were hospitalized during the observation period, the LOS in the intervention group was considerably shorter. A possible explanation is that patients in the intervention group were in more frequent touch with their healthcare workers and could more quickly be discharged back to the FACT Plus programme. Once back in FACT Plus, they could then be monitored more closely. It is also possible that this closer contact resulted in fewer – zero versus nine – compulsory admissions, a subcategory of admissions which indicates high illness severity and low treatment adherence [14, 15].

Comparison of our study with the studies in Germany and England [10, 12] reveals similarities and differences regarding the programmes themselves and their study designs. First, the content of each programme was very similar, focusing on psychoeducation on the psychiatric condition, and also on adherence and preventing rehospitalization. Second, while the English programme was provided to individual patients, our programme and the German programme were provided in groups. Third, while the German programme lasted for 4 to 5 months, the English one was extended to 18 months, and ours lasted for between nine and 12 months. The study designs also differed: while we chose a quasi-experimental non-randomized controlled study, the German study used block randomization to allocate patients to the intervention group or control group, and the English study used a pre-post design. Despite these differences, however, the three studies had comparable results.

### Strengths and limitations

This study included patients who were difficult to engage in treatment and care, and who would probably have been even more difficult to include in research studies. It was designed with a minimum of exclusion criteria, and was carried out in the context of routine daily FACT. At the design stage we considered the advantages and disadvantages of randomization, and particularly wished to avoid selection bias, which would have arisen if any of the most seriously ill patients had not been included because they refused to sign the informed consent form. For this reason we accepted the disadvantage of non-randomized allocation to the intervention group and control group. Despite this, the number of patients enrolled in this pilot study remained rather small and a larger sample size would be required for more definitive answers.

In the sensitivity analyses – which left out 1.) control patients who had were already been admitted at the start of the observation period and 2.) the control group patient had been in hospital for more than 300 days during the observation period – the outcomes with regard to LOS were only moderately attenuated, but did not change essentially. However, the outcomes with regard to costs were considerably attenuated in the sensitivity analyses.

The intervention consisted of a nine-month multimodal group programme with one weekly session. However, as with the preceding German and English studies, its design did not allow us to establish which elements of the intervention were responsible for the effects we observed.

Overall, ours is the third study after its German and English forerunners to test this intensive multimodal group programme. With regard to the reduction in LOS – roughly 50% in all three studies – and in mental healthcare costs, its results are strikingly similar.

## Conclusions

After this intensive multimodal group programme (FACT Plus), patients with psychotic disorders who risked rehospitalization spent 19.4 fewer days (56.1%) in psychiatric hospitalization than patients in care as usual (FACT). This promising result was accompanied by a limited reduction in mental healthcare costs. These findings indicate that FACT Plus, a Dutch adaptation of comparable programmes in Germany and England, may make meaningful improvements to FACT – and possibly to other community mental health teams – for patients with a psychotic disorder who risk rehospitalization. More generally, these findings also indicate that combining an intensified treatment programme with regular mental healthcare in the community may be a valuable add-on for the most vulnerable psychiatric patients risking rehospitalization.

## Data Availability

We are considering follow-up publication with regard to the present data set. Thus, we are not yet ready to make the data publicly available. We are willing, though, to share the data on request.

## Abbreviations

ACT: Assertive community treatment
FACT: Flexible assertive community treatment
LOS: Length of stay

## References

[1] Delespaul PH, de consensusgroep EPA (2013). Consensus regarding the definition of persons with severe mental illness and the number of such persons in the Netherlands. Tijdschr Psychiatr.

[2] Ruggeri M, Leese M, Thornicroft G, Bisoffi G, Tansella M (2000). Definition and prevalence of severe and persistent mental illness. Br J Psychiatry.

[3] Botha UA, Koen L, Joska JA, Parker JS, Horn N, Hering LM (2010). The revolving door phenomenon in psychiatry: comparing low-frequency and high-frequency users of psychiatric inpatient services in a developing country. Soc Psychiatry Psychiatr Epidemiol.

[4] Pfiffner C, Steinert T, Kilian R, Becker T, Frasch K, Eschweiler G (2014). Rehospitalization risk of former voluntary and involuntary patients with schizophrenia. Soc Psychiatry Psychiatr Epidemiol.

[5] Di Lorenzo R, Sagona M, Landi G, Martire L, Piemonte C, Del Giovane C (2016). The revolving door phenomenon in an Italian acute psychiatric ward: a 5-year retrospective analysis of the potential risk factors. J Nerv Ment Dis.

[6] Stein LI, Test MA (1980). Alternative to mental hospital treatment. I. Conceptual model, treatment program, and clinical evaluation. Arch Gen Psychiatry.

[7] Dieterich M, Irving CB, Bergman H, Khokhar MA, Park B, Marshall M (2017). Intensive case management for severe mental illness. Cochrane Database Syst Rev.

[8] van Veldhuizen JR (2007). FACT: a Dutch version of ACT. Community Ment Health J.

[9] Nugter MA, Engelsbel F, Bahler M, Keet R, van Veldhuizen R (2016). Outcomes of flexible assertive community treatment (FACT) implementation: a prospective real life study. Community Ment Health J.

[10] Pitschel-Walz G, Bauml J, Bender W, Engel RR, Wagner M, Kissling W (2006). Psychoeducation and compliance in the treatment of schizophrenia: results of the Munich psychosis information project study. J Clin Psychiatry.

[11] Bauml J, Pitschel-Walz G, Volz A, Luscher S, Rentrop M, Kissling W (2016). Psychoeducation improves compliance and outcome in schizophrenia without an increase of adverse side effects: a 7-year follow-up of the Munich PIP-study. Schizophr Bull.

[12] Lewis L, O'Keeffe C, Smyth I, Mallalieu J, Baldock L, Oliver S (2016). Maintaining adherence Programme: evaluation of an innovative service model. BJPsych Bull.

[13] American Psychiatric Association. Diagnostic and statistical manual of mental disorders (4th edn) (DSM-IV). American Psychiatric Association, 1994.

[14] de Jong MH, Kamperman AM, Oorschot M, Priebe S, Bramer W, van de Sande R (2016). Interventions to reduce compulsory psychiatric admissions: a systematic review and meta-analysis. JAMA Psychiatry.

[15] de Jong MH, Oorschot M, Kamperman AM, Brussaard PE, Knijff EM, van de Sande R (2017). Crucial factors preceding compulsory psychiatric admission: a qualitative patient-record study. BMC Psychiatry.

